# Complete genome sequences of two *Bacillus thuringiensis* serovar *kurstaki* strains isolated from Lebanon and Tunisia, highly toxic against lepidopteran larvae

**DOI:** 10.1128/MRA.00060-23

**Published:** 2023-08-08

**Authors:** Nancy Fayad, Rita Barssoum, Nathalie Marsaud, Rayan Nasseredine, Nouha Abdelmalek, Souad Rouis, Marie Ange Teste, Vincent Pailler, Veronique Gautier, Elodie Belmonte, César Arturo Aceves Lara, Julien Cescut, Luc Fillaudeau, Mireille Kallassy Awad

**Affiliations:** 1 Laboratory of Biodiversity and Functional Genomics, Université Saint-Joseph de Beyrouth, Beirut, Lebanon; 2 Multi-Omics Laboratory, School of Pharmacy, Lebanese American University, Byblos, Lebanon; 3 Toulouse Biotechnology Institute, Toulouse, France; 4 GenoToul GeT-BioPUCE, Toulouse, France; 5 Laboratoires Pharmaceutiques MédiS, Tunis, Tunisia; 6 Laboratory of Biopesticides, Centre of Biotechnology of Sfax, University of Sfax, Sfax, Tunisia; 7 Gentyane, GDEC, INRAE, Clermont-Ferrand, France; 8 Toulouse White Biotechnology, Toulouse, France; University of Southern California, Los Angeles, California, USA

**Keywords:** *Bacillus thuringiensis*, genomes, whole-genome sequencing, PacBio

## Abstract

*Bacillus thuringiensis*-based products are key in the biopesticides market. *Bacillus thuringiensis kurstaki* strains Lip and BLB1 were isolated from Lebanese and Tunisian soils, respectively. These strains are highly toxic against lepidopteran larvae, *Ephestia kuehniella*. Here, we report Lip and BLB1 complete genomes, including their plasmid and toxin contents.

## ANNOUNCEMENT

*Bacillus thuringiensis* serovar *kurstaki* (*Btk*) strains Lip and BLB1 were isolated from Lebanese and Tunisia soil samples, respectively (1 - 3). Both strains showed an increased toxicity against the lepidopteran *Ephestia kuehniella* larvae in comparison to the reference strain HD-1. Their entomopathogenic potential was evaluated by *in vivo* toxicity assays and showed a lethal concentration of 50% of the larvae (LC50) of 33.27 and 70 ng of toxin per milligram of flour for Lip and BLB1, respectively (1 - 3). In the context of IPM-4-Citrus (MSCA RISE, No. 734921, 2017–2023), a project aiming to optimize the culture of these strains on a wheat bran-based medium, a whole-genome sequencing (WGS) approach was adopted to elucidate all genomic aspects of *Btk* Lip and BLB1.

For WGS, strains were grown in liquid Luria-Bertani (LB) medium for 16 h at 30°C, after which DNA was extracted using the Monarch HMW DNA Extraction Kit as per manufacturer instructions. WGS was conducted using a PacBio Sequel II Sequencer (Pacific Biosciences, Menlo Park, CA, USA) (4, 5) at the Gentyane platform (Clermont-Ferrand, France). For PacBio sequencing, library was prepared using a SMRTbell prep kit3, following manufacturer instructions. Genomic DNA was sheared, cleaned of single-strand overhangs, repaired for damage and A A tailed. Then, barcoded overhang adapters were ligated to generate the SMRTBell templates. Fragments above 5 kb were then size selected with 35% AMPure PB Beads. A Fragment Analyzer (Agilent Technologies) and a Qubit fluorimeter (Life Technologies) allowed quantity and quality checks. A ready-to-sequence SMRTBell Polymerase Complex was created using a Binding Kit 3.2 (PacBio) and the Sequel II primer 3.2. A consensus sequencing (CCS) mode was adopted, and reads were later demultiplexed with Bam2fastx software (Bioconda) under default parameters.

The number of CCS reads was 524,791 for Lip and 374,689 for BLB1 (Table 1). Assembly was done with flye v2.5, under default parameters (6). Overlaps were manually removed. The final coverage was 417.3× and 746.6× for Lip and BLB1, respectively. Genome completeness was assessed by BUSCO v5.0, using default parameters (7), and was found to be at 99.78% for Lip and 96% for BLB1. Circular contigs were first highlighted by the Flye assembler repeat graphs and then further identified as plasmids via a database similarity search using a nucleotide BLAST+ (2.12.0+ [8]) executable *blastn* command. This allowed to search for identified plasmids against the complete *Bacillus thuringiensis* genomes from NCBI assembly (NCBI:txid1428). Additional checks of completeness, identity, and circularity of plasmids were done by multiple sequence alignment comparison with several reference *Btk* strains: HD-1 (ASM71753v1 [9]), YBT-1520 (ASM74754v1), and HD73 (ASM33875v1 [10]).

**TABLE 1:** Sequence features and accession numbers of replicons from *Btk* strains Lip and BLB1

	Replicon	Bioproject/biosample/NCBI SRA accession	Accession number	Length (bp)	CDS # (total)	# Cry coding genes	GC content (%)	Number of PacBio CCS reads/reads N50
**Lip**	Chromosome	PRJNA924104/SAMN32746259/SRX20261752	CP116313	5,293,947	5,632		35.2	524,791/9,650
	pLip2189		CP116316	2,189	4		35.3	
	pLip7635		CP116320	7,635	8		32.2	
	pLip7911		CP116321	7,911	9		32.3	
	pLip8513		CP116322	8,513	9		30.8	
	pLip12		CP116314	12,276	21		31.1	
	pLip15		CP116315	15,008	28		34.9	
	pLip69		CP116319	69,004	76		32.2	
	pLip91		CP116323	91,357	102		31.2	
	pLip97		CP116324	97,437	85	2: Cry1Ab; Cry1Ac	34.5	
	pLip300		CP116317	300,451	267	5: Cry1Aa; Cry1Ac; Cry1Ia; Cry2Aa; Cry2Ab;	33.1	
	pLip457		CP116318	457,481	408		32.7	
**BLB1**	Chromosome	PRJNA924104/SAMN32746260/SRX20261751	CP116325	5,677,911	6,060		35.3	374,689/8,967
	pBLB1_7792		CP116333	7,792	9		32.3	
	pBLB1_8398		CP116335	8,398	10		29.8	
	pBLB1_8548		CP116336	8,548	8		30.8	
	pBLB1_12		CP116326	11,521	9	1: Cry1Ab	40.3	
	pBLB1_14		CP116327	14,174	21		32.5	
	pBLB1_15		CP116328	15,071	22		31.2	
	pBLB1_48		CP116330	47,643	73		35.4	
	pBLB1_56		CP116331	56,399	62		32.2	
	pBLB1_74		CP116332	73,558	88		30.6	
	pBLB1_81		CP116334	80,698	86		33.5	
	pBLB1_317		CP116329	317,321	282	5: Cry1Aa; Cry1Ac; Cry1Ia; Cry2Aa; Cry2Ab;	33.2	

Each strain carried 11 different plasmids ranging between 2 and 457 kb. pLip15 was identified as a linear tectiviral prophage, quite similar to GIL16, a tectivirus also isolated from a *Bacillus thuringiensis* strain (11).

Genome annotation was done with NCBI’s automated annotation pipeline Prokaryotic Genome Annotation Pipeline (PGAP) (12). Toxin content was mined using t.BLAST.n with the toxin protein sequences recovered from the Bacterial Pesticidal Protein Resource Center (https://www.bpprc-db.org [13]). Genes encoding toxins from the Cry1 and Cry2 families were detected in Lip on plasmids pLip300 and pLip97, and BLB1 on pBLB1_317 and pBLB1_12 (Table 1).

## Data Availability

Whole-genome sequences for *Bacillus thuringiensis* serovar *kurstaki* strains Lip and BLB1 are available in NCBI’s GenBank under the bioproject number PRJNA924104 for both strains and the accession numbers CP116313 to CP116324 for Lip and CP116325 to CP116336 for BLB1. Raw reads are also available on the NCBI SRA database under the bioproject number PRJNA924104.
