# Erratum for Fayad et al., “Complete genome sequences of two *Bacillus thuringiensis* serovar *kurstaki* strains isolated from Lebanon and Tunisia, highly toxic against lepidopteran larvae”

**DOI:** 10.1128/mra.00694-24

**Published:** 2024-08-20

**Authors:** Nancy Fayad, Rita Barssoum, Nathalie Marsaud, Rayan Nasseredine, Nouha Abdelmalek, Souad Rouis, Marie Ange Teste, Vincent Pailler, Veronique Gautier, Elodie Belmonte, César Arturo Aceves Lara, Julien Cescut, Luc Fillaudeau, Mireille Kallassy Awad

## ERRATUM

Volume 12, no. 9 , e00060-23, 2023, https://doi.org/10.1128/mra.00060-23. Page 2, Table 1: In row 21, "Cry1Ab" should read "Cry1Aa."

